# Polyvinylidene Fluoride-Based Gel Polymer Electrolytes for Calcium Ion Conduction: A Study of the Influence of Salt Concentration and Drying Temperature on Coordination Environment and Ionic Conductivity

**DOI:** 10.1021/acs.jpcc.3c02342

**Published:** 2023-08-15

**Authors:** Edward C. Fluker, Shreyas Pathreeker, Ian D. Hosein

**Affiliations:** Syracuse University, 329 Link Hall, Syracuse, New York 13244, United States

## Abstract

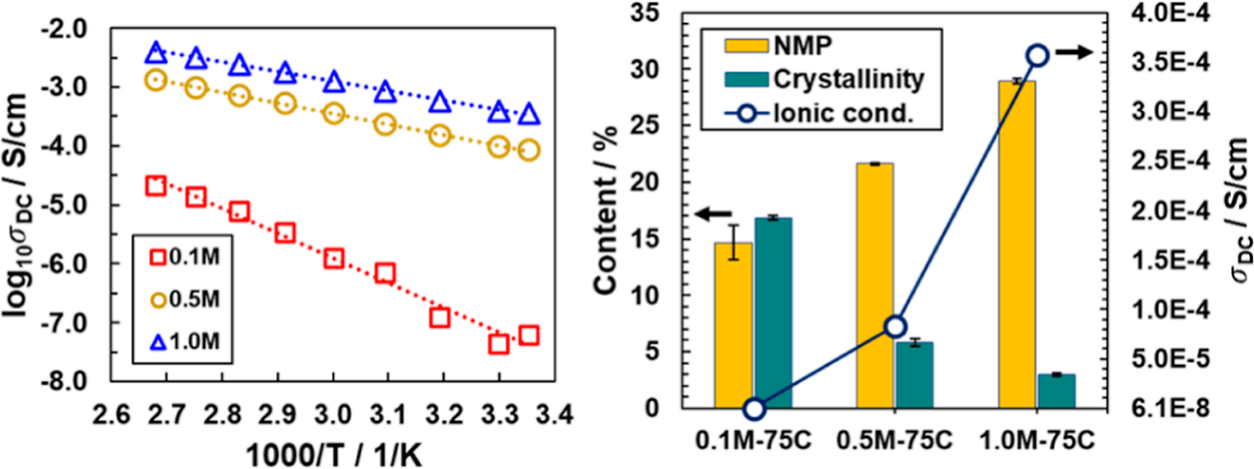

Calcium-ion batteries emerged as a potential sustainable alternative energy storage system; however, there remains the need to further develop electrolytes to improve their performance. We report a gel polymer electrolyte (GPE)-based on polyvinylidene fluoride (PVDF) for calcium ion conduction. The gel electrolyte was synthesized by combining a PVDF polymer host, Ca(TFSI)_2_ salt, and *N*-methyl-2-pyrrolidone (NMP) solvent. Using Fourier transform infrared spectroscopy, we analyze the effect of salt concentration and drying temperature on the degree of salt dissociation in the electrolyte. Our results show that the concentration of free cations in the electrolyte is primarily coordinated with NMP as well as PVDF, generating a suitable network for ion transport, i.e., a liquid electrolyte encompassed within a polymer matrix. We find that processing conditions such as drying temperature, which varies solvent content, play a critical role in developing polymer electrolytes that demonstrate optimal electrochemical performance. The GPEs are semicrystalline and stable up to 120 °C, which is critical for their use in applications such as in electric vehicles and renewable energy storage systems. The ionic conductivity of the GPEs exhibit Arrhenius-type behavior, and the total ionic conductivity at room temperature is suitable for applications, with values of 0.85 × 10^–4^ S/cm for 0.5 M and 3.56 × 10^–4^ S/cm for 1.0 M concentrations. The results indicate that the GPE exhibits high conductivity and good stability, making it a promising candidate for use in high-performance calcium ion batteries.

## Introduction

In recent years, there has been a growing demand for high-performance and sustainable electrochemical energy storage systems, particularly for portable electronic devices, vehicle electrification, and stationary energy storage. Lithium-ion systems are at the forefront of state-of-the-art energy storage technologies to meet this ever-increasing demand. However, the limited supply of lithium and its potential environmental impacts have led to an increasing interest in exploring alternative battery chemistries based on safer and more earth abundant elements. Calcium-ion batteries provide several advantages over lithium-ion batteries, including their high theoretical capacity, similar redox potential to lithium, natural abundance, and lower cost.^1^ Despite these advantages, the commercialization of calcium-ion batteries is still limited by several challenges, including the development of stable and efficient electrolytes.^2^

Polymer electrolytes have been extensively studied for their application in various battery systems such as Li-, Na-, and Mg-ion batteries. However, despite the advantages of Ca-ion batteries as a potential sustainable alternative, there are only limited reports on the development of polymer electrolyte systems for Ca-ion batteries. This highlights the need for further research and development of polymer electrolytes tailored specifically for Ca-ion batteries to improve their electrochemical performance and promote their commercialization as a viable energy storage system. Polymer electrolytes have shown great promise in enhancing the performance of various battery chemistries, including Ca-ion batteries, by offering several advantages such as thermal stability, electrochemical stability, and suitable conductivities.^2^ Therefore, the development of suitable polymer electrolytes for Ca-ion batteries could significantly contribute to the development of sustainable and efficient energy storage systems. To date, only polyether polymer backbone systems containing ether functionalities have been explored for use in calcium-ion batteries. However, the strong complexation and coordination of calcium ions to the ether groups in the polymer backbone can lead to low ionic conductivities and issues with the kinetics of electrochemistry at the electrodes. Therefore, it is desirable to explore other types of polymer backbone chemistries and polymer compositions to investigate possible enhancements in calcium-ion conductivity. By altering the coordination strength of the calcium to the functional units of the polymer, it may be possible to overcome the limitations of the polyether systems. This is particularly important, as the divalent nature of calcium ions makes them highly coordinated to ether functions in the polymer, which can affect the overall performance of the battery.

Gel polymer electrolytes (GPEs), on the other hand, have shown enormous promise for use in calcium-ion batteries due to their numerous additional benefits relative to solid polymer electrolytes. High ionic conductivity GPEs are associated with transport within the liquid fraction of the electrolyte, which also provides good wetting against the electrodes thereby enabling promising electrochemical kinetics. Additionally, polymer gel electrolytes exhibit good thermal stability and electrochemical stability. The most significant advantage of using a polymer gel electrolyte in calcium-ion batteries is the opportunity for calcium to coordinate with the liquid phase of the gel electrolyte, which facilitates liquid-like conductivity within the polymer host. Since calcium ions prefer to coordinate with the liquid electrolyte, minimal coordination with the polymer backbone may occur, making the system polymer backbone agnostic. This provides a degree of freedom to engineer polymer host chemistry to improve other properties such as mechanical and thermal properties. To date, only two gel systems have been explored for calcium-ion batteries, both using poly(ethylene glycol diacrylate) (PEGDA) as the backbone and either an alkyl carbonate electrolyte or an ionic liquid electrolyte.^3,4^ Both systems show high conductivity, as well as thermal, mechanical, and electrochemical stability, and reasonable electrochemical kinetics. However, there still remains a rich polymer chemistry space previously examined for other cation systems that involve different polymer backbones and solvents to explore potential improvements in the properties of gel electrolytes. Therefore, investigating alternative compositions can provide additional insight into the design and optimization of polymer gel electrolytes for calcium-ion batteries, which can ultimately lead to the development of more efficient and sustainable Ca-based energy storage systems.

Polyvinylidene fluoride (PVDF) has emerged as a promising polymer backbone for use in both dry polymer electrolytes and GPEs in various battery systems. PVDF offers several advantages, including high thermal stability, good mechanical strength, and a high dielectric constant, which enhances the electrolyte’s conductivity and battery performance. Furthermore, PVDF is compatible with a wide range of electrode materials, such as lithium, sodium, magnesium, and zinc, making it suitable for use in different battery systems. PVDF also resists chemical degradation by the liquid electrolyte, which helps increase the battery’s overall lifespan. Hence, PVDF is a highly desirable polymer backbone for use in dry polymer electrolytes or GPEs in energy storage systems. Due to these properties, PVDF has found use in emerging multivalent chemistries such as Mg with promising results at both the material-level as well as the cell-level. Deivanayagam and coworkers^5^ have reported a reasonable room temperature ionic conductivity (RTIC) of 0.16 mS/cm using PVDF-co-HFP composite membranes and Mg(ClO_4_)_2_ salt. Shortly thereafter, Singh et al. developed electrospun PVDF-co-HFP-based gel electrolytes with impressive ionic conductivity of 1.62 mS/cm.^6^ However, there are no reports on the electrolyte properties of a PVDF-based gel electrolyte for Ca-batteries.

Herein, we report the development of a GPE based on PVDF. The gel electrolyte was synthesized by combining PVDF host, Ca(TFSI)_2_ salt, and *n*-methyl-2-pyrrolidone (NMP) solvent. Ca(TFSI)_2_ was chosen due to its low lattice energy, large anion size, and delocalized anion charge that synergistically promote salt dissociation that is desirable for high ionic conductivity. NMP solvent was chosen due to its polarity and ability to dissolve both the PVDF and the salt. We analyzed the effect of salt concentration and drying temperature on the degree of salt dissociation in the electrolyte using Fourier transform infrared (FTIR) spectroscopy. The results show that the concentration of free cations in the electrolyte is primarily coordinated with NMP as well as PVDF, generating a suitable network for ion transport. We also highlight the critical role of processing conditions, such as drying temperature, in developing GPEs with optimal electrochemical performance. The GPEs demonstrate excellent thermal stability and high ionic conductivity, making them a promising candidate for use in high-performance calcium-ion batteries. The ionic conductivity of the GPEs exhibited Arrhenius-type behavior, and the total ionic conductivity at room temperature was 0.85 × 10^–4^ S/cm for 0.5 M and 3.56 × 10^–4^ S/cm for 1.0 M salt concentrations which is suitable for application in energy storage devices. Hence, this work provides valuable insights into the development of polymer electrolytes for calcium-ion batteries, which can help overcome the existing challenges and promote its commercialization as an alternative, sustainable energy storage system.

## Materials and Methods

### Materials

Battery grade PVDF (600,000 g/mol) was purchased from MTI Corporation and used as received. Ca(TFSI)_2_ salt was purchased from Sigma-Aldrich, USA. The salt was stored in an argon filled glovebox with moisture and oxygen levels <0.5 ppm. Polymer solutions were prepared by dissolving 1.25 g of PVDF in 20 mL NMP in the ambient and stirred inside the glovebox for 1 h and dried over molecular sieves for 24 h. Electrolyte solutions with salt concentrations of 0.1, 0.5, and 1.0 M were prepared inside the glovebox by dissolving appropriate amounts of Ca(TFSI)_2_ salt in 10 mL NMP. The electrolyte solutions were stirred for 1 h and dried over molecular sieves for 24 h. For investigating the influence of drying temperature on ionic conductivity, the polymer solution and 0.5 M electrolyte solution were mixed in a 1:1 ratio by volume, stirred for an hour, and then drop-cast onto glass slides to dry. The drying temperature was either 75, 95, or 115 °C in the glovebox on a hot plate, and the drying time was 24 h. For investigating the influence of salt concentration on ionic conductivity, 0.1, 0.5, and 1.0 M electrolyte solutions were each mixed with the polymer solution in a 1:1 ratio by volume and stirred for 1 h. Solutions were then drop-cast onto clean glass slides to dry at 75 °C for 24 h on a hot plate in the glovebox.

### Spectroscopy

FTIR spectroscopy was carried out in attenuated total reflectance (ATR) mode using a Nicolet iS5 spectrometer at a resolution of 4 cm^–1^ with 16 scans per collection. Background subtraction, normalization, and peak deconvolution of the spectra were performed using the OriginLab Pro software package.

### Polymer Characterization

Thermogravimetric analysis (TGA) of the polymer samples was carried out in platinum pans using a TA Instruments Q500 analyzer under N_2_ flow from room temperature to 600 °C at a ramp rate of 10 °C/min. Differential scanning calorimetry (DSC) was carried out using a TA Instruments Q200 calorimeter between −50 and 200 °C. For both TGA and DSC measurements, sample weights were between 1.5 and 8 mg.

### Electrochemical Characterization

Electrochemical impedance spectroscopy was carried out using a Solartron EnergyLabXM instrument with stainless steel blocking electrodes at an amplitude of 10 mV and in the frequency range of 1 MHz to 0.1 Hz. Spectra were first collected at room temperature and then between 30 and 70 °C in steps of 10 °C. An 18 minute soak was applied to the test cells at each temperature to ensure thermal equilibration of the polymer samples. Ionic conductivities were calculated using the following equation:
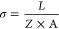
where *L* is the thickness of polymer samples (in the range of 300–650 μm), *A* is the area of the polymer samples, and *Z* is the real part of the impedance obtained from impedance spectroscopy. The length and area were measured for each sample using a micrometer.

## Results and Discussion

**Figure 1:**
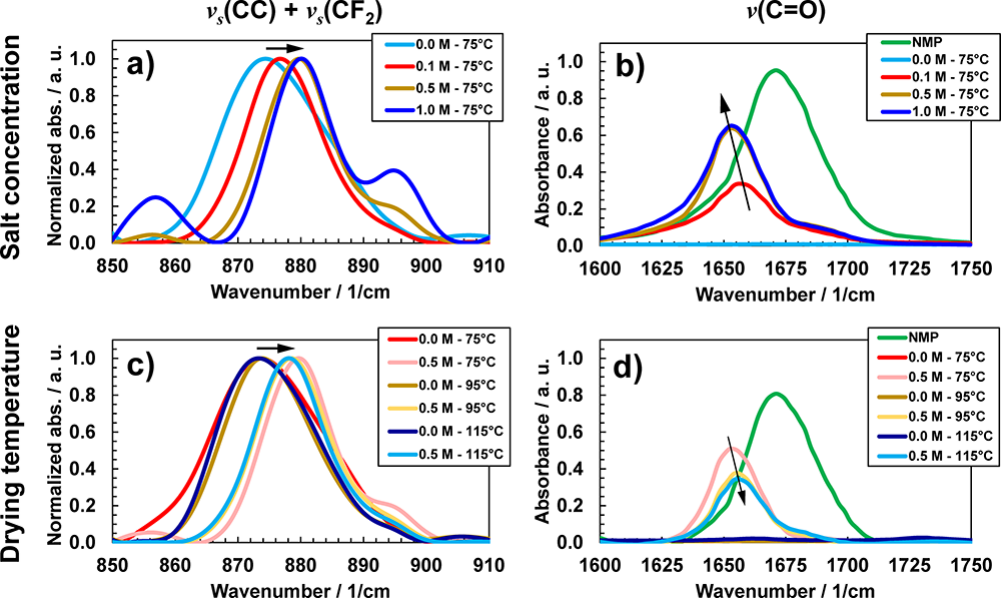
FTIR spectra of PVDF (first column) and NMP (second column) for (a, b) varying salt concentrations and a fixed drying temperature of 75 °C, and (c, d) NMP with varying drying temperatures and a fixed salt concentration of 0.5 M. The respective vibrational modes are indicated above the plots.

We first employed FTIR spectroscopy to elucidate the solvation environment of the calcium ions. PVDF and NMP can both contribute to salt dissociation and subsequent coordination with Ca^2+^ cations due to the 3 lone electron pairs on the F atom in PVDF and the single lone electron pair on the O atom in NMP. Shifts in their characteristic FTIR peaks would be associated with PVDF–NMP, NMP–Ca^2+^, and PVDF–Ca^2+^ interactions. Peak assignments can be found in Tables S1–S3 in the Supporting Information. Figure 1a shows FTIR spectra for the combined C–C bond symmetric stretching and CF_2_ bond symmetric stretching of the PVDF backbone.^7^ The neat PVDF film (i.e., in the absence of salt) demonstrates a peak at 875 cm^–1^ that shifts to higher wavenumbers upon addition of salt, which indicates a change in the coordination environment associated to PVDF. Shifts in the C–C vibrational band as seen in Figure 1a must by necessity be as a result of coordination occurring with the C–F bond, as the latter is the only possible coordinating chemical function on PVDF. The 875 cm^–1^ peak shift is greater with increased salt concentration, indicating that the wavenumber shift is salt concentration dependent, i.e., with higher the salt concentration, the greater the change is in the Ca^2+^ coordination environment with PVDF. Besides this main PVDF FTIR peak, two satellite peaks arise at 855 and 893 cm^–1^ upon addition of salt and become stronger in peak intensity with increase in salt concentration. The peak at 855 cm^–1^ also exists as a small, broad peak in pure NMP solvent, and as such is indexed to NMP. The peak at 893 cm^–1^ overlaps with neither NMP nor the salt and, therefore, must originate from a second population (i.e., phase) of PVDF in the GPEs. To provide context, a few studies on the piezoelectric properties of PVDF have attributed this shoulder peak at 893 cm^–1^ to the β-phase of PVDF.^7,8^ Yet, the GPE films herein are neither mechanically stretched nor subjected to a large electric field—steps known and applied previously to reorient the polymer crystals via induction of net dipoles along the PVDF chains. Also, in a previous study, Lee and coworkers have reported that the addition of CaF_2_ particles to PVDF promoted the formation of polymer crystalline phases.^9^ Whether doping with salt can “activate”/“induce” the β-phase of PVDF is unclear, and is the focus of further investigation.^10^

In Figure 1b, FTIR spectra are shown for the primary peak of NMP solvent originating from C=O stretching band.^11^ Relative to neat NMP solvent whose characteristic peak is at 1670 cm^–1^, the GPEs demonstrate a large downward shift of this NMP peak (order of ∼15 cm^–1^). This shift may be attributed to coordination of NMP with the salt cation and interaction with PVDF. The data also suggests a correlation between the presence of salt in the polymer and NMP retention in the GPEs. Namely, when salt is absent (i.e., a 0.0 M PVDF–NMP system), the neat PVDF film does not exhibit the typical NMP peak (light blue line in Figure 1b). Figure 1a,b demonstrate a shift in both the PVDF and NMP peaks with an increase in salt concentration. This shift can be attributed to the simultaneous increase in salt and solvent content, as the salt retains the solvent (which will be discussed later).

Despite the use of concentration-varied FTIR spectroscopy, it remains unclear to what extent the shift in peaks is due to PVDF–NMP, PVDF–Ca^2+^, or NMP–Ca^2+^ interactions. In order to delineate between these interactions, we first began by examining GPEs with 0.0 M and 0.5 M salt concentrations and varied the NMP liquid fraction via sample drying at different temperatures (75, 95, and 115 °C), i.e., higher temperatures yield lower NMP content. Both salt and no-salt polymer systems were otherwise prepared using identical procedures. We note that the salt concentration range explored in this work was guided by studies on liquid electrolytes containing Ca(TFSI)_2_ salt, wherein the highest ionic conductivities were obtained in the range of 0.5 M and 1.0 M salt concentration, beyond which the ionic conductivity decreased considerably due to ion aggregation.^12^ Furthermore, this salt concentration range is sufficient for battery application. Furthermore, at drying temperatures below 75 °C, freestanding polymer films could not be obtained. The lowest drying temperature, therefore, was 75 °C. As shown in Figure 1c, the vibrational mode of PVDF at ∼875 cm^–1^ shows the same large ∼15 cm^–1^ shift to higher wavenumbers, as observed in Figure 1a, when salt is included in the system (i.e., PVDF–NMP–Ca^2+^ at 0.5 M vs PVDF–NMP with 0.0 M). Increases in the drying temperature for the 0.5 M system only mildly shift the peak further to higher wavenumbers, and there is no shift observed for the PVDF–NMP (0 M) system with increase in drying temperature. Hence, the combination of the results shown Figure 1a,c would indicate that the shifts in the PVDF vibrational mode are associated with PVDF–Ca^2+^ interactions. We similarly examined the NMP peak for samples with 0.0 M and 0.5 M salt concentrations, and varied sample drying temperatures, as shown in Figure 1d. All peaks at 0.0 M have extremely low peak intensities relative to the 0.5 M samples (appears as almost flat lines in the spectra of Figure 1d). The NMP peak intensities for 0.5 M samples decrease as the drying temperature increases, suggesting that a decrease in NMP content with higher drying temperature results in weaker interactions with Ca^2+^ ions. Since the spectral positions for PVDF remain unaffected by the drying temperature, but the NMP peak shifts with drying temperature (which is associated with a reduction in NMP content), it is likely that the Ca^2+^ cation coordination environment is more closely linked to interactions with the NMP solvent.

Initially the upshift in the NMP peak with increasing drying temperature may seem counterintuitive because, with increasing drying temperature, there is less free NMP, leading to the possibility of more NMP coordination with calcium ions. Indeed, the incorporation of salt results in coordination-induced retention of solvent in the electrolyte at the molecular level, and thus ability of the calcium salt to retain more NMP content suggests that, with more NMP drying, only Ca^2+^-coordinated NMP remains in the system, and a downward shift in the NMP peak is expected. Later, as an explanation for the results, we provide evidence for the emergence of ion-pairing in the GPEs as an explanation for the observed changes in NMP peaks.

Collectively, the data in Figure 1 data reveal a coordination environment within the GPEs consisting of Ca^2+^–NMP interactions and Ca^2+^–PVDF interactions. Indeed, we can infer preference for coordination of salt with NMP over PVDF owning in part to the higher dielectric strength of NMP (ε_r_ = 32)^13^ compared to that of PVDF (ε_r_ = 8),^14^ which implies that NMP is more effective at dissociating the salt than is the PVDF polymer matrix. NMP is a good solvent for PVDF (calculated solubility parameter χ = 0.0013), which makes NMP–PVDF a popular solvent-binder system in the preparation of anode and cathode films for batteries, and similar interactions between small molecule solvents and PVDF have been reported in the literature in the context of GPEs.^15−17^

**Figure 2:**
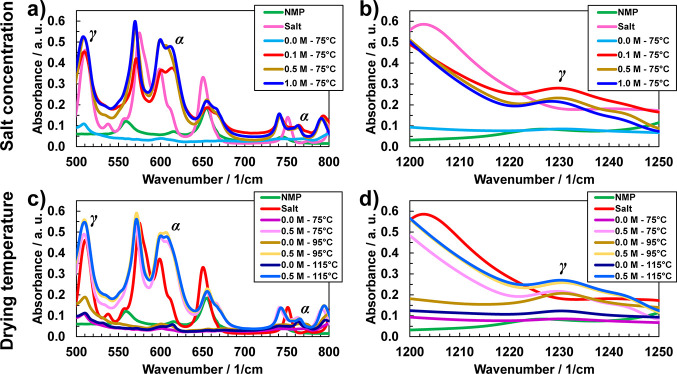
FTIR spectra indicating the α- and γ- phases of PVDF for (a,b) different salt concentrations and a fixed drying temperature of 75 °C, and (c,d) for different drying temperatures and a fixed salt concentration of 0.5 M.

In regard to the PVDF structure, the polymorphism of PVDF crystals plays an important role in its ability to coordinate with cations.^18^ PVDF exists in several crystalline phases, namely, α-, β-, and γ-,^19^ of which the α phase is the most common and consists of alternating CF_2_ groups arranged opposite to one another on the main chain. This structural configuration of α-PVDF renders it non-polar, and, therefore, PVDF–Ca^2+^ complexes would not generally be expected. Yet, the F atoms in PVDF carry lone pairs available for dative bonding, and evidence of complex formation between PVDF and salt cations has indeed been presented previously. For example, Jacob et al. investigated the effect of PEO addition to PVDF–LiClO_4_ mixtures and used XRD analysis to confirm PVDF–Li^+^ interactions,^20^ Chiang et al. used XPS analysis to confirm PVDF–Li^+^ interactions.^21^ Shen et al. reported PVDF–Li^+^ coordination based on vibrational spectroscopy data.^22^ More recently, however, Mathies et al.^23^ in their work using a PVDF–HFP matrix for Li-ion conduction suggest that ion transport in the PVDF polymer matrix occurs via molecular-level channels formed by TFSI^–^ aggregates due to the non-coordinating nature of PVDF. On the other hand, β-PVDF and γ-PVDF are known to be electroactive due to the CF_2_ groups being aligned, thereby, generating a directional dipole. Cai et al.^19^ have noted via rigorous analysis of FTIR data that peaks in the range of 840 and 510 cm^–1^ may be indicative of combined β-PVDF and γ-PVDF phases if there are also peaks present at ∼1275 and ∼1234 cm^–1^, respectively. Additionally, the presence of the α-PVDF can also be confirmed by the presence of peaks at ∼614 and ∼763 cm^–1^. Notably, characteristic peaks originating from α-PVDF and γ-PVDF are found in the GPEs as shown in Figure 2 in the spectral regions of 510 to 840 (α and γ phases, Figure 2a,c) and in the spectral region from 1200 to 1250 cm^–1^ (γ phase, Figure 2b,d) These two phases persist in samples over all concentrations and drying temperatures explored herein (also shown in Figure 2). Based on this analysis, our GPEs comprise the non-electroactive α-PVDF and electroactive γ-PVDF, with the presence of the latter providing further corroboration (owing to its own peak shifts across salt concentration and drying temperature) of the PVDF interactions in the GPE systems, as shown in Figure 1.

**Figure 3:**
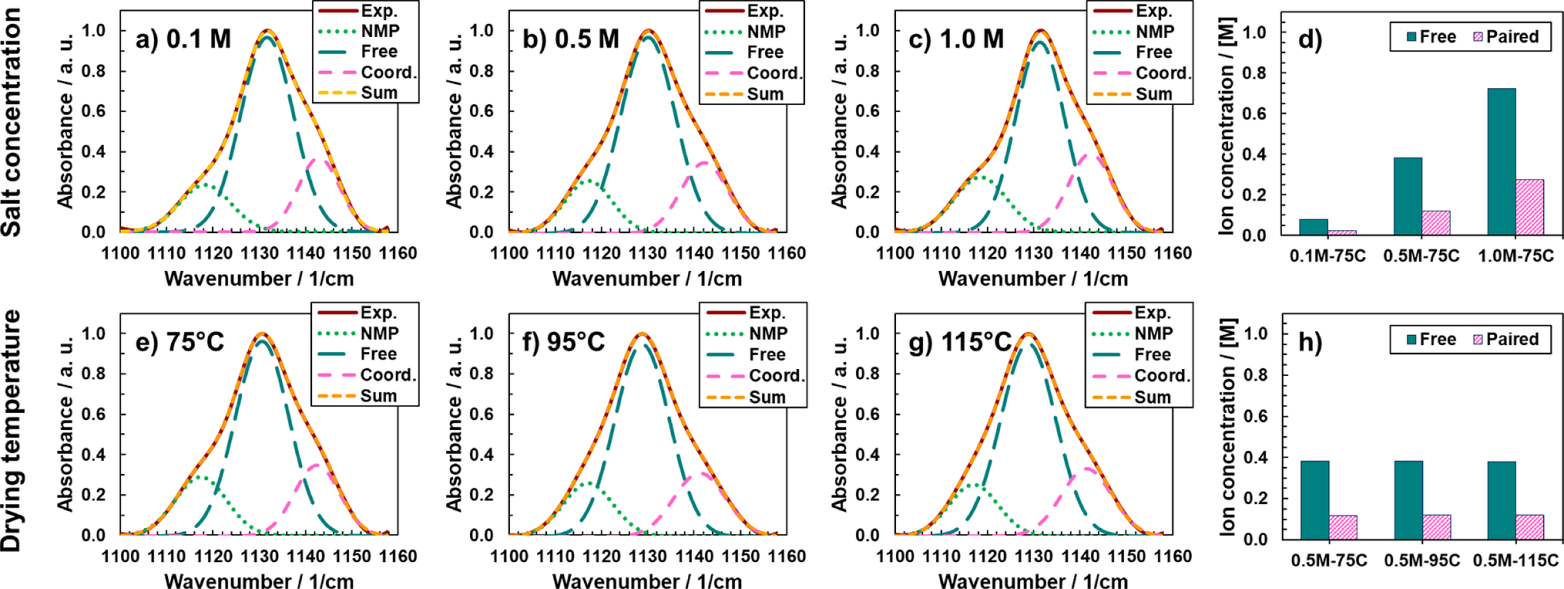
Deconvoluted FTIR spectra of the O=S=O vibration of TFSI^–^ anion. The spectral profiles are fit to three Gaussian peaks, associated to the NMP, free anions, and ion pairs (i.e., coordinated). (a–c) FTIR spectra analysis for different salt concentrations at a fixed drying temperature of 75 °C. (d) Calculated concentrations of the free anions and ion pairs respective salt concentrations shown in (a–c). (d–f) FTIR spectra analysis for different drying temperatures at a fixed salt concentration of 0.5 M. (h) calculated concentrations of the free anions and ion pairs respective salt concentrations shown in (e–g).

To gain a more comprehensive understanding of the coordination environment within the GPEs, we conducted in-depth FTIR peak analysis to determine the composition of the various coordinating species, particularly in the liquid fraction (i.e., the solvent environment). FTIR spectral regions associated to the salt anion (TFSI^–^) were deconvoluted to decouple free and paired anion species. Figure 3a–c show deconvoluted data for the three salt concentrations explored. The spectral region associated to the TFSI^–^ anion was deconvoluted into three separate peaks and their attributable species: the shoulder peak at ∼1120 cm^–1^ attributed to pure NMP and is, therefore, not considered in this analysis, the primary peak at ∼1130 cm^–1^ attributed to “free” or unpaired TFSI^–^ ions, and the shoulder peak at ∼1142 cm^–1^ originating from the formation of TFSI^–^ ion pairs.^2,24^Figure 3d–f show deconvoluted FTIR spectra for the TFSI^–^ anion, once again as a function of drying temperature at a fixed salt concentration of 0.5 M. The concurrent presence of the primary peak (1130 cm^–1^) and the shoulder peak (1142 cm^–1^) indicates, respectively, the presence of free anions and paired anions in the GPEs, over all concentrations and drying temperatures explored. Full spectrum FTIR data are available in the Supporting Information (Figures S1 and S2).

Figure 3d,h presents a summary of the concentrations of free anions and ion pairs, respectively. These values were obtained by integrating the fitted Gaussian peak areas in Figure 3a–c,e–g and plotted as a function of concentration (for the lowest drying temperature, 75 °C) and drying temperature (for salt concentration of 0.5 M). As expected, an increase in salt concentration is accompanied by a rise in the concentration of free anions and ion pairs (Figure 3d). Accounting for the 2:1 relationship between anion (TFSI^–^) and cation (Ca^2+^), the concentration of free (i.e., dissociated) cations in the electrolyte would be half of the value for the free anions. This fraction of dissociated cations is primarily coordinated/solvated with NMP and possibly PVDF within the gel electrolyte, creating a solid polymer matrix that encompasses dissociated salt in a liquid solvent medium, thereby generating a suitable network for ion transport. As shown in Figure 3h, the concentration of free anions and ion pairs remains relatively constant across all drying temperatures, most likely due to the salt’s ability to retain NMP and suppress its evaporation, especially since the drying temperatures are below the boiling point of NMP (∼202 °C). This constant distribution of free anions and paired anions was observed for all salt concentrations, with the fraction of ion pair concentration being greater for higher salt concentrations (i.e., 1.0 M vs 0.5 and 0.1 M at the same drying temperature).

**Figure 4:**
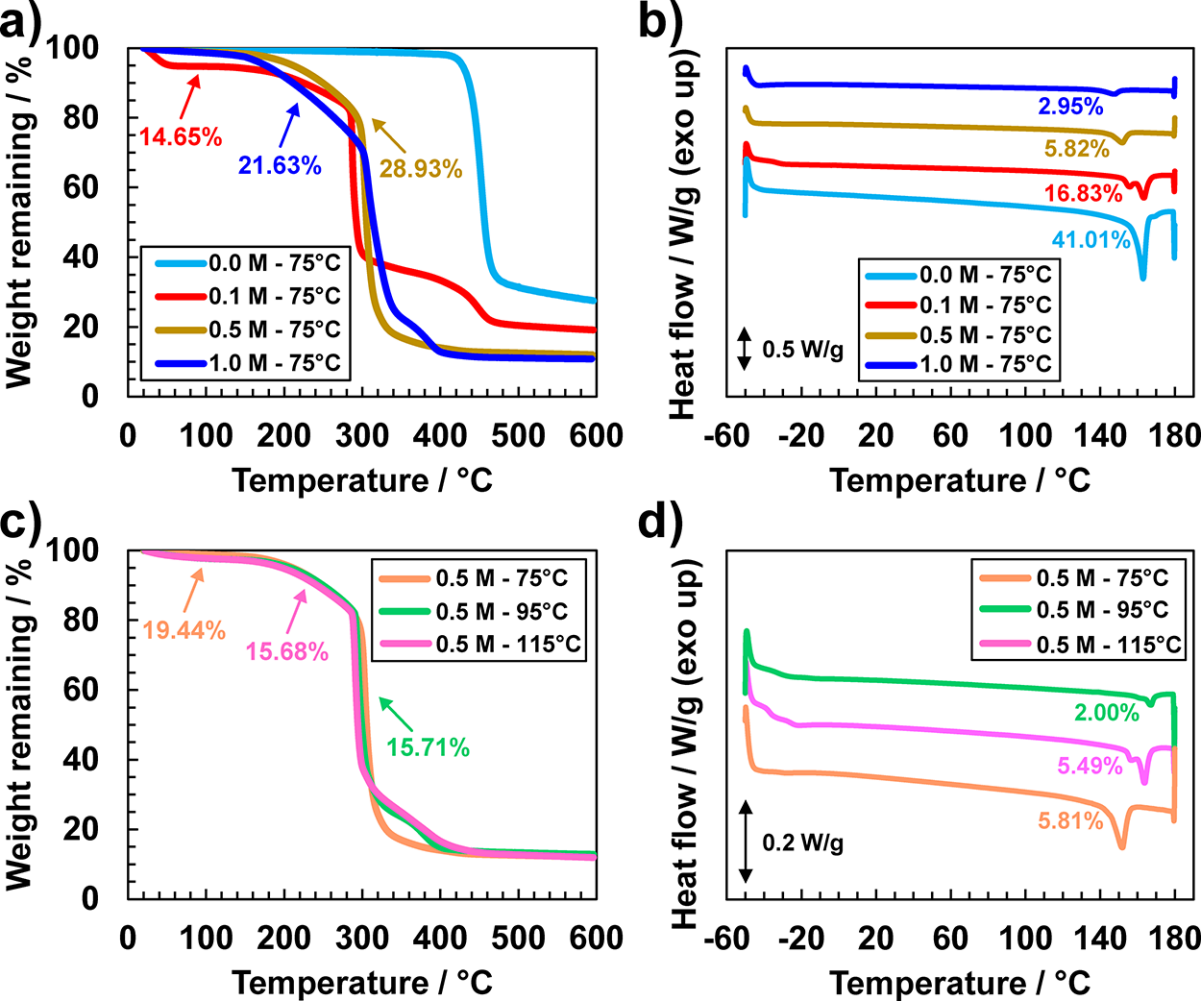
TGA traces and DSC traces for (a,b) different salt concentrations and a fixed drying temperature of 75 °C, and (c,d) NMP for different drying temperatures and a fixed salt concentration of 0.5 M. Arrows in (a,c) indicate the NMP content in weight %, whereas numbers in (c,d) indicate the percent crystallinity of the samples.

Thermal characterization of the polymer electrolytes was carried out using TGA and DSC. As shown in Figure 4a, the thermal stability of the polymer electrolytes varies considerably with salt concentration. A pure PVDF film prepared identically to the electrolyte samples remains thermally stable up to 450 °C, which agrees well with thermal studies conducted previously.^21,25,26^ Note that for this pure PVDF film, mass loss from solvent evaporation is negligible (nearly flat line until polymer degradation). In contrast, the degradation profile of the polymer electrolytes (i.e., in the presence of salt) is more gradual and occurs over a broad temperature range with an onset temperature of 120 °C, and a sharp mass loss detected at 300 °C. We attribute the gradual mass loss to evaporation of bound (i.e., coordinated) NMP molecules from the electrolytes, whereas the sharp mass loss at 300 °C is associated to the thermal degradation of PVDF. Wei and coworkers have also reported that NMP degrades over a temperature range of 110 to 270 °C.^27^ We observed that the thermal stability of the PVDF polymer electrolytes decreases as salt concentration increases, as shown in Figure 4a. This accelerated degradation of PVDF in the polymer electrolytes, compared to pure PVDF, is likely due to its interactions with NMP and possibly Ca(TFSI)_2_, which either plasticizes PVDF, or suppresses its crystallinity, or both.

After polymer degradation, remnant mass is highest for pure PVDF, followed by 0.1, 0.5, and 1.0 M, indicating that more PVDF has degraded in the polymer electrolytes relative to pure PVDF. Changes to the structure of PVDF upon addition of salt reflect in the DSC heating traces shown in Figure 4b. While pure PVDF melts at 163 °C, the GPEs exhibit decreasing melting temperatures with increasing salt concentration. The melting temperature (*T*_m_) values for the GPEs are 156 °C (0.1 M) > 152 °C (0.5 M) > 147 °C (1.0 M), i.e., the melting point of PVDF decreases with increasing salt concentration due to its interactions with NMP and the salt. Note that for 0.1 M, two peaks are detected—a main peak at 163 °C, and a satellite peak at 156 °C, which we denote as the *T*_m_ for this sample. Because the main peak overlaps with that for pure PVDF, the smaller peak must originate from a different population (i.e., phase) of PVDF in the electrolyte that is more accessible to melting. Finally, the glass transition temperature (*T*_g_) of pure PVDF could not be detected here possibly owing to a premature cut-off temperature during the DSC scans. However, the *T*_g_ for the polymer electrolytes can be detected at approximately −35 °C for 0.1 and 0.5 M, whereas *T*_g_ for 1.0 M is not discernible likely due to a high heating rate. The low *T*_g_ values observed in our GPEs further confirm the suitability of PVDF as a polymer matrix for Ca-ion conduction.

As shown in Figure 4c, the degradation profiles for samples with the same salt concentration but dried at different temperatures overlap considerably with one another, following the same two-step degradation of solvent followed by polymer as seen in Figure 4a. However, interesting findings emerge from the DSC traces for these samples dried at different temperatures (but same salt concentration of 0.5 M), enabling us to decouple the effect of salt and the effect of solvent on the structure of the polymer. As shown in Figure 4d, the lowest *T*_m_ is observed for the drying temperature of 75 °C, whereas the highest melting point is observed for the drying temperature of 95 °C. Surprisingly, the sample dried at 115 °C exhibits two melting peaks—one at 163 °C that overlaps with the *T*_m_ of pure PVDF, and a satellite peak at 160 °C, which can be attributed to a second phase of PVDF solvated by the solvent, and possibly coordinated with Ca^2+^. This feature is strikingly similar to that observed for 0.1 M in Figure 4b and can be explained based on the content of NMP in the polymer electrolytes. The non-normalized FTIR spectra for the polymer electrolytes in Figure 1 clearly indicate a lower (and similar) NMP peak intensity for 0.1 M, 75 °C and 0.5 M, 115 °C samples. The DSC traces for these two samples also appear to be similar, suggesting that the drying temperature counteracts the effect of higher salt concentration by decreasing the amount of NMP in the polymer electrolyte. Lastly, *T*_g_ for the polymer electrolytes dried at different temperatures consistently falls around −30 °C. In summary, we find that the GPEs developed herein are semicrystalline due to the presence of both a *T*_g_ and a *T*_m_ and are stable up to a temperature of at least 120 °C, which is sufficient for battery operation. We also find that the degradation profiles of the GPEs are indifferent to the drying temperature at a fixed salt concentration. The increased mass loss prior to the onset of PVDF degradation with increase in salt concentration indicates the increase in NMP content in the GPEs associated to that higher salt concentration, i.e., salt helps retain solvent in the GPEs.

**Figure 5:**
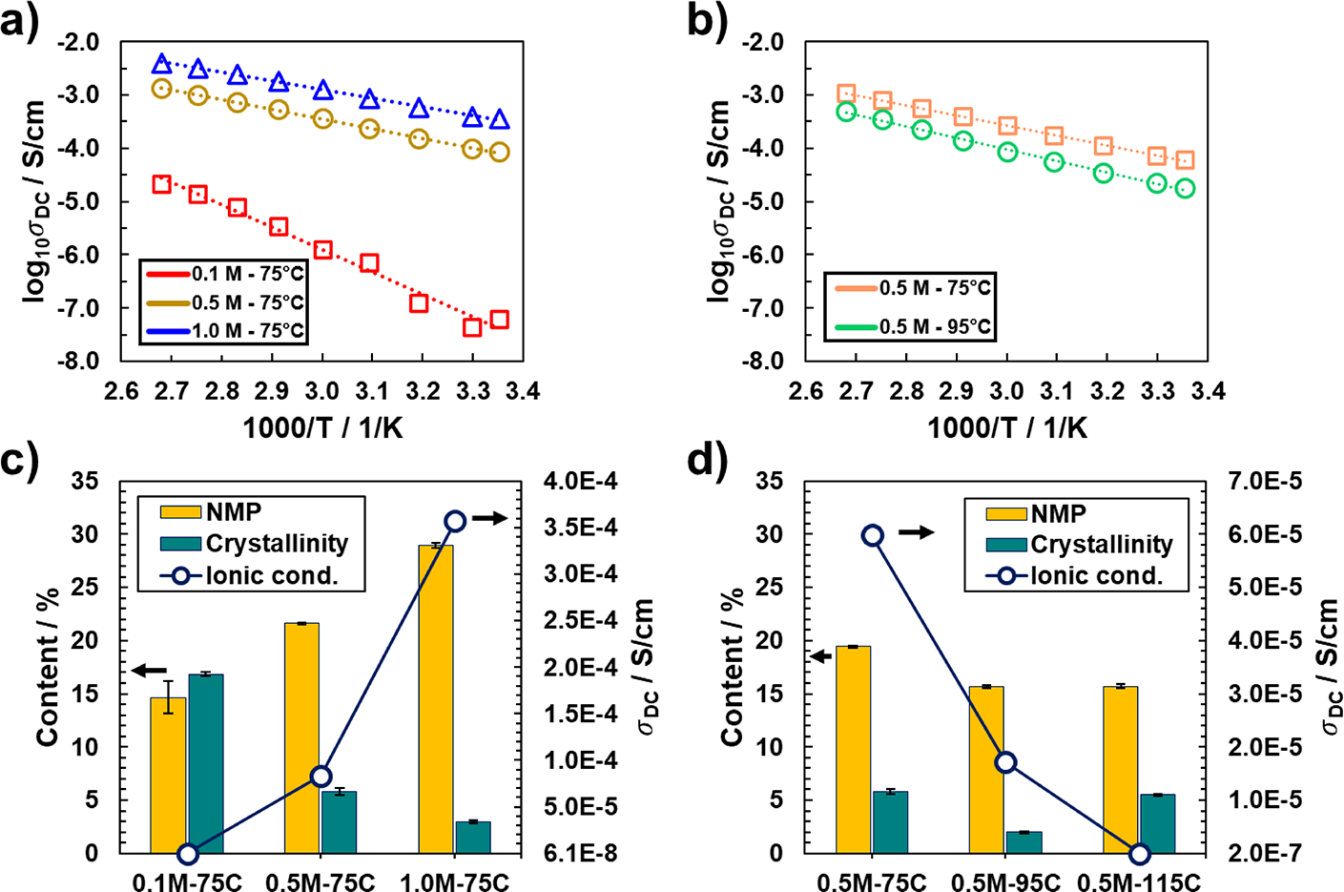
Arrhenius plots for (a) different salt concentrations and a fixed drying temperature of 75 °C, and (b) different drying temperatures and a fixed salt concentration of 0.5 M; TGA-derived NMP content (left *y*-axis) and measured ionic conductivity (right *y*-axis) as a function of (c) salt concentration (drying temperature of 75 °C), and (d) drying temperature (salt concentration of 0.5 M). In (c,d), error bars for % NMP represent one standard deviation of the average of 4 NMP content calculations using different temperature ranges on a single sample, and error bars for % crystallinity represent one standard deviation of the average of 3 crystallinity calculations using different temperature ranges on a single sample. Note that the *y*-axes in (c,d) are modified to capture the lowest ionic conductivity.

For ionic conductivity studies, raw impedances were extracted from the frequency-independent plateau of real impedance versus frequency plots (see Figures S3 and S4, Supporting Information). Figure 5a,b shows Arrhenius plots of ionic conductivity vs inverse temperature across salt concentration and drying temperature, respectively. The plots show that increasing the salt concentration leads to an increase in total ionic conductivity, which is in accordance with the charge carrier concentration-dependent nature of ionic conductivity.^28^ We note that this behavior is also due to the higher fraction of free charge carriers as shown in Figure 3 from FTIR analysis. In Figure 5a, the ionic conductivity of the 0.1 M GPE is observed to be ∼10^–8^ S/cm at room temperature, but a sharp increase in total ionic conductivity (nearly 4 orders of magnitude) is observed from 0.1 to 0.5 M salt concentrations. Ion transport is influenced by the presence of solvent in the polymer matrix due to differences in viscosity and dielectric strength between the two phases, and the ability of solvents to plasticize the polymer matrix. This is indicated in Figure 5b, which shows a drop in the ionic conductivity curve for GPEs dried at 95 vs 75 °C. The conductivity of the GPE dried at 115 °C could not be reliably fitted across different temperatures, most likely owing to its very low conductivity.^29^

The correlations to and effects of drying temperature discussed thus far are primarily linked to the NMP content in the system, which is varied via the drying temperature. Since our FTIR analysis reveals a direct correlation between the presence of salt and the presence of NMP, to quantify the amount of NMP present in the GPEs, we analyzed the mass loss from 100 to ∼280 °C (from the TGA data). Figure 5c,d plot the NMP content and the ionic conductivity as a function of salt concentration. The TGA-derived NMP content in the GPEs increases with increase in salt concentration, as does the ionic conductivity. The increase in ionic conductivity is therefore due to a combined effect of increased charge carrier concentration as well as a consequent (and necessary) higher NMP content which is coordinated to the salt and facilitates faster ion motion (and greater carrier flux) owing to a lower GPE viscosity and increased carrier mobility. Charge carrier mobility is related to the activation energy, which we find to be lower for the higher salt (and consequently higher solvent) concentration based on the slopes of the Arrhenius plot shown in Figure 5a (dashed lines). For different drying temperatures at a fixed salt concentration of 0.5 M, we observed a reduction in NMP content from 22% at 75 °C to 17% at 95 °C, which corresponds to a decrease in the total ionic conductivity of the sample by approximately 3-fold. As shown in Figure 5b, we found that the ionic conductivity of 0.5 M–75C was 0.60 × 10^–4^ S/cm, whereas a slightly lower ionic conductivity of 0.17 × 10^–5^ S/cm was observed for 0.5 M–95C, which is due to lower NMP content in the GPE. To place this into context, similar behavior has been reported previously by Yao and coworkers^26^ for PVDF–Li electrolyte. At the highest drying temperature of 115 °C, the NMP content derived from TGA analysis was similar to that at 95 °C, but the ionic conductivity dropped to about 2 × 10^–7^ S/cm. Note that this value is similar to that obtained for the 0.1 M sample dried at 75 °C. One plausible reason for this behavior could be the structure of PVDF, which is indicated in the FTIR data in Figure 1 and in the DSC curve in Figure 4d. Only a small fraction of PVDF is likely coordinated with the salt in this particular composition, which may affect the number of ion pairs observed in the FTIR analysis. However, we did not notice any significant differences in FTIR data for the TFSI^–^ anion represented by O=S=O vibrations. Careful analysis of representative Bode plots (Supporting Information) reveals that the sample dried at 115 °C exhibits apparent plateaus of real impedance in the low frequency region compared to all the other samples that exhibit plateaus in the middle frequency to high frequency regions that shift to lower impedances in accordance with decreased relaxation times with increase in temperature. More importantly, the data in this region are noisy, which prevents the determination of a reliable ionic conductivity value. It is possible that structural changes to PVDF occur due to the high drying temperature (for example, changes in crystalline phases of PVDF, reorientation of crystal lamellae, and so forth),^23^ which we are currently investigating. To reveal the effect of structure on the conductivity properties, Figure 5c,d also provide the % degree of crystallinity of the PVDF samples, determined from the DSC traces. The degree of crystallinity decreases with increased salt concentration, as expected due to the salt and solvent in the systems providing significant PVDF–NMP and PVDF–Ca^2+^ interactions to reduce polymer crystallinity. Interestingly, samples dried at 115 °C have a slightly higher crystallinity, and, thus, the expected greater quantity of crystallites in the GPE can impede ion motion, thus to some extent explaining the drop in ionic conductivity. It is also possible that morphologically, at this high drying temperature, the remaining NMP solvent might exist in a noncontinuous form, with NMP solvent possibly having phase segregated within the polymer host. Both the additional crystallinity and possible discontinuity of the NMP phase may explain the drastic reduction in ionic conductivity, despite having a similar NMP content at GPEs dried at lower temperatures. Our findings highlight the critical role of processing conditions, such as drying temperature, in developing polymer electrolytes with suitable gel morphology to obtain optimal charge carrier transport. Further investigations may be necessary to better understand the underlying mechanism for the observed behavior, and the associated molecular-scale morphology of the PVDF and solvent phases in the GPEs.

Overall, our data demonstrate a linear dependence of ionic conductivity on inverse temperature, suggesting Arrhenius-type behavior. This linearity indicates that despite the physically rubbery nature of the polymer matrix, contribution to ion transport from polymer segmental motion is small owing to the much higher dissociating ability of NMP solvent. Therefore, ion transport occurs primarily via hopping between coordinating sites or by vehicular motion, as seen in pure liquid electrolytes. This finding is supported by the primary coordination of Ca^2+^ with NMP as revealed by FTIR analysis, as well as the semicrystalline nature of PVDF observed in the DSC traces.

To the best of our knowledge, this is the first report investigating semicrystalline PVDF matrices for Ca ion conduction. Our group has previously investigated Ca-ion conduction in poly(ethylene glycol) (PEG)-based cross-linked polymer frameworks containing different solvents. Using 50 wt % ethylene carbonate (EC) and Ca(TFSI)_2_ salt, we observed a room temperature ionic conductivity (RTIC) on the order of 10^–5^ S/cm,^3^ whereas using 50% [EMIM][Otf] ionic liquid and Ca(TFSI)_2_ salt led to a higher RTIC on the order of 10^–4^ S/cm.^4^ The RTIC of the PVDF-based gel electrolyte reported here is higher than its PEG-based amorphous gel counterparts while employing much lesser solvent. Notably, the highest ionic conductivity found here for the salt concentration of 1.0 M (dried at 75 °C), 0.35 mS/cm, is higher than all other polymer electrolytes reported in the literature for Ca ion conduction (solid and gel),^2^ with the exception of our previous findings with a vinylimidazole-based gel electrolyte that demonstrates a RTIC in the vicinity of 1 mS/cm.^2^ Overall, our findings demonstrate the potential of semicrystalline PVDF hosts to realize high-performance electrolytes for Ca ion conduction.

## Conclusions

In summary, we have presented the utilization of PVDF-based GPEs for calcium ion conduction, an area that has not been previously investigated. We reveal the dual coordination environment for Ca^2+^ (with the solvent and polymer) based on changes in the vibrational modes of both PVDF and NMP. The primary coordinating species for the salt cation is NMP, which is supported by Arrhenius plots of ionic conductivity that demonstrate a linear trend with inverse temperature. In addition to salt concentration, we also investigate the effect of drying temperature on polymer electrolyte properties and ionic conductivity. For a fixed drying temperature of 75 °C, the total ionic conductivity of the polymer electrolytes increases with increase in salt concentration owing not only to a larger number of charge carriers but also due to the presence of more NMP in the electrolyte with more salt. On the other hand, at a fixed salt concentration of 0.5 M, increasing the drying temperature leads to a reduction in NMP content and changes in PVDF crystallinity, which collectively decrease ionic conductivity. These findings reflect the importance of NMP (or solvent content in general) in a PVDF-based GPE system with regard to Ca^2+^ conductivity. Ionic conductivity depends strongly on not only salt concentration but also the NMP content retained in the GPE, which depends on the drying conditions. The ionic conductivities obtained in this study are in the range of 10^–4^ S/cm, with the highest ionic conductivity being 0.35 mS/cm. This promising result motivates cell-level investigation, which is ongoing. Further structural investigation using scattering techniques is necessary to fully understand the role of PVDF and gel morphology on the observed behavior of the GPEs. In addition, investigating different polymer backbones, such as polytetrahydrofuran (PTHF), polyimide (PI), and polyacrylonitrile (PAN) (three hosts that have yet to be thoroughly explored for calcium), may offer valuable insights into their coordination functions and densities for calcium ion conduction based on their different chemistries and coordinating strengths. We also note that the effect of solvents on ion transport in gel polymer electrolytes also remains underexplored and can also be the focus of future work. This could significantly advance our general understanding of the polymer and gel properties for calcium ion systems.

## Supporting Information Available

Full spectrum FTIR data for different salt concentrations; full spectrum FTIR data for different drying temperatures; representative Bode plots for different salt concentrations; representative Bode plots for different drying temperatures; FTIR peak assignment for NMP solvent; FTIR peak assignments for Ca(TFSI)2 salt; and FTIR peak assignments for PVDF polymer (PDF)

## References

[ref1] Hosein I. D. The promise of calcium batteries: open perspectives and fair comparisons. ACS Energy Lett. 2021, 6, 1560–1565. 10.1021/acsenergylett.1c00593.

[ref2] Pathreeker S.; Hosein I. D. Vinylimidazole-Based Polymer Electrolytes with Superior Conductivity and Promising Electrochemical Performance for Calcium Batteries. ACS Appl. Polym. Mater. 2022, 4, 6803–6811. 10.1021/acsapm.2c01140. 36277173 PMC9578112

[ref3] Biria S.; Pathreeker S.; Genier F. S.; Chen F. H.; Li H.; Burdin C. V.; Hosein I. D. Gel Polymer Electrolytes Based on Cross-Linked Poly (ethylene glycol) Diacrylate for Calcium-Ion Conduction. ACS Omega 2021, 6, 17095–17102. 10.1021/acsomega.1c02312. 34250366 PMC8264931

[ref4] Biria S.; Pathreeker S.; Genier F. S.; Hosein I. D. A highly conductive and thermally stable ionic liquid gel electrolyte for calcium-ion batteries. ACS Appl. Polym. Mater. 2020, 2, 2111–2118. 10.1021/acsapm.9b01223.

[ref5] Deivanayagam R.; Cheng M.; Wang M.; Vasudevan V.; Foroozan T.; Medhekar N. V.; Shahbazian-Yassar R. Composite polymer electrolyte for highly cyclable room-temperature solid-state magnesium batteries. ACS Appl. Energy Mater. 2019, 2, 7980–7990. 10.1021/acsaem.9b01455.

[ref6] Singh R.; Janakiraman S.; Agrawal A.; Ghosh S.; Venimadhav A.; Biswas K. An amorphous poly (vinylidene fluoride-co-hexafluoropropylene) based gel polymer electrolyte for magnesium ion battery. J. Electroanal. Chem. 2020, 858, 113788 10.1016/j.jelechem.2019.113788.

[ref7] Chowdhury F. I.; Khandaker M. U.; Amin Y. M.; Kufian M. Z.; Woo H. J. Vibrational, electrical, and structural properties of PVDF–LiBOB solid polymer electrolyte with high electrochemical potential window. Ionics 2017, 23, 275–284. 10.1007/s11581-016-1857-0.

[ref8] Peng H. K.; Wu M. M.; Wang Y. T.; Li T. T.; Sun F.; Lou C. W.; Lin J. H. Enhancing piezoelectricity of poly (vinylidene fluoride) nano-wrapped yarns with an innovative yarn electrospinning technique. Polym. Int. 2021, 70, 851–859. 10.1002/pi.6177.

[ref9] Eun J. H.; Sung S. M.; Kim M. S.; Choi B. K.; Lee J. S. Effect of MWCNT content on the mechanical and piezoelectric properties of PVDF nanofibers. Mater. Des. 2021, 206, 109785 10.1016/j.matdes.2021.109785.

[ref10] Lee S. G.; Ha J. W.; Sohn E. H.; Park I. J.; Lee S. B. Enhancement of polar crystalline phase formation in transparent PVDF-CaF_2_ composite films. Appl. Surf. Sci. 2016, 390, 339–345. 10.1016/j.apsusc.2016.08.090.

[ref11] Ponzio E. A.; Echevarria R.; Morales G. M.; Barbero C. Removal of N-methylpyrrolidone hydrogen-bonded to polyaniline free-standing films by protonation–deprotonation cycles or thermal heating. Polym. Int. 2001, 50, 1180–1185. 10.1002/pi.755.

[ref12] Forero-Saboya J. D.; Marchante E.; Araujo R. B.; Monti D.; Johansson P.; Ponrouch A. Cation solvation and physicochemical properties of Ca battery electrolytes. J. Phys. Chem. C 2019, 123, 29524–29532. 10.1021/acs.jpcc.9b07308. PMC6961307 31956392

[ref13] George J.; Sastry N. V. Densities, viscosities, speeds of sound, and relative permittivities for water+ cyclic amides (2-pyrrolidinone, 1-methyl-2-pyrrolidinone, and 1-vinyl-2-pyrrolidinone) at different temperatures. J. Chem. Eng. Data 2004, 49, 235–242. 10.1021/je0340809.

[ref14] Luo B.; Wang X.; Wang Y.; Li L. Fabrication, characterization, properties and theoretical analysis of ceramic/PVDF composite flexible films with high dielectric constant and low dielectric loss. J. Mater. Chem. A 2014, 2, 510–519. 10.1039/C3TA14107A.

[ref15] Periasamy P.; Tatsumi K.; Shikano M.; Fujieda T.; Saito Y.; Sakai T.; Mizuhata M.; Kajinami A.; Deki S. Studies on PVdF-based gel polymer electrolytes. J. Power Sources 2000, 88, 269–273. 10.1016/S0378-7753(99)00348-1.

[ref16] Zhang M. Y.; Li M. X.; Chang Z.; Wang Y. F.; Gao J.; Zhu Y. S.; Wu Y.; Huang W. A sandwich PVDF/HEC/PVDF gel polymer electrolyte for lithium ion battery. Electrochim. Acta 2017, 245, 752–759. 10.1016/j.electacta.2017.05.154.

[ref17] Jie J.; Liu Y.; Cong L.; Zhang B.; Lu W.; Zhang X.; Liu J.; Xie H.; Sun L. High-performance PVDF-HFP based gel polymer electrolyte with a safe solvent in Li metal polymer battery. J. Energy Chem. 2020, 49, 80–88. 10.1016/j.jechem.2020.01.019.

[ref18] Li C. Y. The rise of semicrystalline polymers and why are they still interesting. Polymer 2020, 211, 123150 10.1016/j.polymer.2020.123150.

[ref19] Cai X.; Lei T.; Sun D.; Lin L. A critical analysis of the α, β and γ phases in poly(vinylidene fluoride) using FTIR. RSC Adv. 2017, 7, 15382–15389. 10.1039/C7RA01267E.

[ref20] Jacob M. M. E.; Prabaharan S. R. S.; Radhakrishna S. Effect of PEO addition on the electrolytic and thermal properties of PVDF-LiClO_4_ polymer electrolytes. Solid State Ionics 1997, 104, 267–276. 10.1016/S0167-2738(97)00422-0.

[ref21] Chiang C. Y.; Shen Y. J.; Reddy M. J.; Chu P. P. Complexation of poly (vinylidene fluoride): LiPF_6_ solid polymer electrolyte with enhanced ion conduction in ‘wet’form. J. Power Sources 2003, 123, 222–229. 10.1016/S0378-7753(03)00514-7.

[ref22] Shen Y. J.; Reddy M. J.; Chu P. P. Porous PVDF with LiClO_4_ complex as ‘solid’and ‘wet’polymer electrolyte. Solid State Ionics 2004, 175, 747–750. 10.1016/j.ssi.2003.10.020.

[ref23] Mathies L.; Diddens D.; Dong D.; Bedrov D.; Leipner H. Transport mechanism of lithium ions in non-coordinating P(VdF-HFP) copolymer matrix. Solid State Ionics 2020, 357, 115497 10.1016/j.ssi.2020.115497.

[ref24] Wen S.; Richardson T. J.; Ghantous D. I.; Striebel K. A.; Ross P.; Cairns E. J. FTIR characterization of PEO+LiN(CF_3_SO_2_)_2_ electrolytes. J. Electroanal. Chem. 1996, 408, 113–118. 10.1016/0022-0728(96)04536-6.

[ref25] Zhang X.; Liu T.; Zhang S.; Huang X.; Xu B.; Lin Y.; Xu B.; Li L.; Nan C. W.; Shen Y. Synergistic coupling between Li_6.75_La_3_Zr_1.75_Ta_0.25_O_12_ and poly (vinylidene fluoride) induces high ionic conductivity, mechanical strength, and thermal stability of solid composite electrolytes. J. Am. Chem. Soc. 2017, 139, 13779–13785. 10.1021/jacs.7b06364. 28898065

[ref26] Yao P.; Zhu B.; Zhai H.; Liao X.; Zhu Y.; Xu W.; Cheng Q.; Jayyosi C.; Li Z.; Zhu J.; Yang Y. PVDF/palygorskite nanowire composite electrolyte for 4 V rechargeable lithium batteries with high energy density. Nano Lett. 2018, 18, 6113–6120. 10.1021/acs.nanolett.8b01421. 30169958

[ref27] Wei Y.; Jang G. W.; Hsueh K. F.; Scherr E. M.; MacDiarmid A. G.; Epstein A. J. Thermal transitions and mechanical properties of films of chemically prepared polyaniline. Polymer 1992, 33, 314–322. 10.1016/0032-3861(92)90988-9.

[ref28] Park B.; Schaefer J. L. Review—Polymer Electrolytes for Magnesium Batteries: Forging Away from Analogs of Lithium Polymer Electrolytes and Towards the Rechargeable Magnesium Metal Polymer Battery. J. Electrochem. Soc. 2020, 167, 070545 10.1149/1945-7111/ab7c71.

[ref29] Gregorio Jr R.; Ueno E. M. Effect of crystalline phase, orientation and temperature on the dielectric properties of poly (vinylidene fluoride)(PVDF). J. Mater. Sci. 1999, 34, 4489–4500. 10.1023/A:1004689205706.

