# Supplementary material for: Polyvinylidene Fluoride-Based Gel Polymer Electrolytes for Calcium Ion Conduction: A Study of the Influence of Salt Concentration and Drying Temperature on Coordination Environment and Ionic Conductivity
Source: J Phys Chem C Nanomater Interfaces. 2023 Aug 15;127(33):16579–87. doi: 10.1021/acs.jpcc.3c02342 (PMC10461727; doi:10.1021/acs.jpcc.3c02342)
Supplement: Supplementary file 1 — jp3c02342_si_001.pdf [file jp3c02342_si_001.pdf]

SUPPORTING INFORMATION

Polyvinylidene Fluoride (PVDF)–Based Gel Polymer Electrolytes For Calcium Ion Conduction: A Study Of The Influence Of Salt Concentration And Drying Temperature On Ionic Conductivity.

Edward C. Fluker,[1] Shreyas Pathreeker,[1] Ian D. Hosein[1*]

1. Syracuse University, 329 Link Hall, Syracuse NY, 13244

Correspondence: idhosein@syr.edu

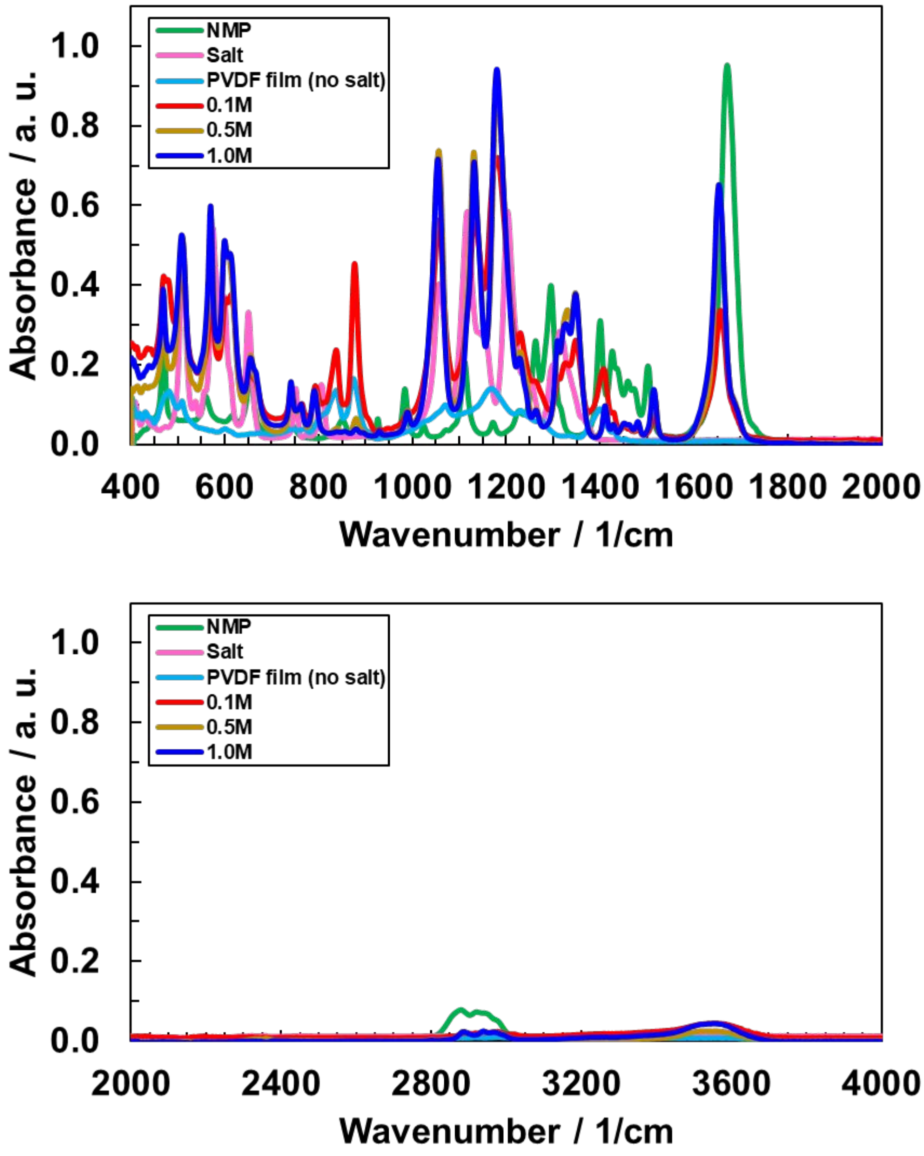

Figure S1. Full spectrum FTIR data for different salt concentrations (75°C drying temperature).

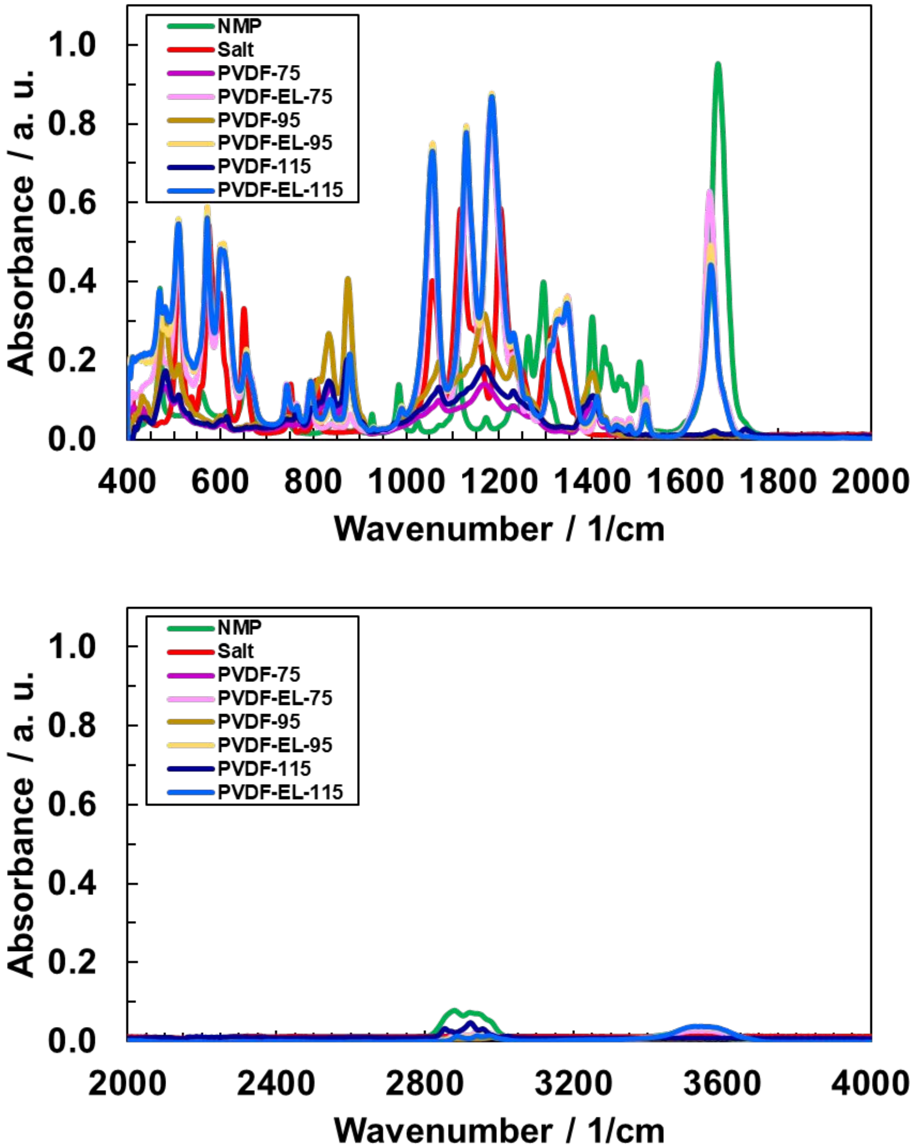

Figure S2. Full spectrum FTIR data for different drying temperatures (0.5M salt concentration).

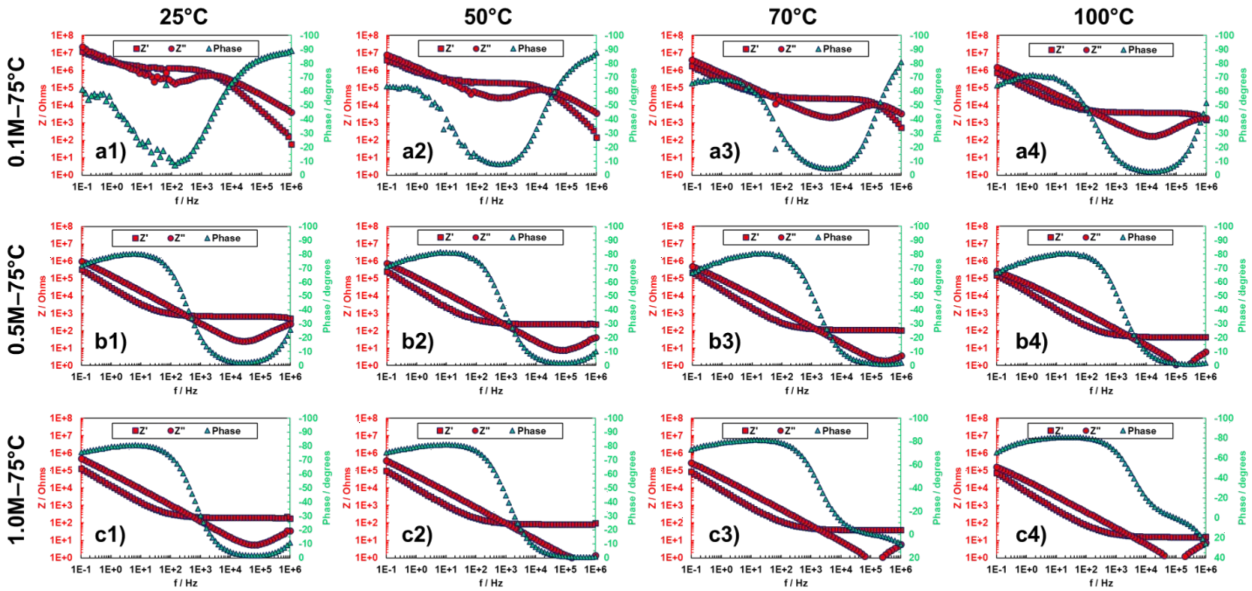

Figure S3. Representative Bode plots for different salt concentrations (75°C drying temperature). Rows represent the salt concentration, and columns represent selected temperatures at which the spectra were collected.

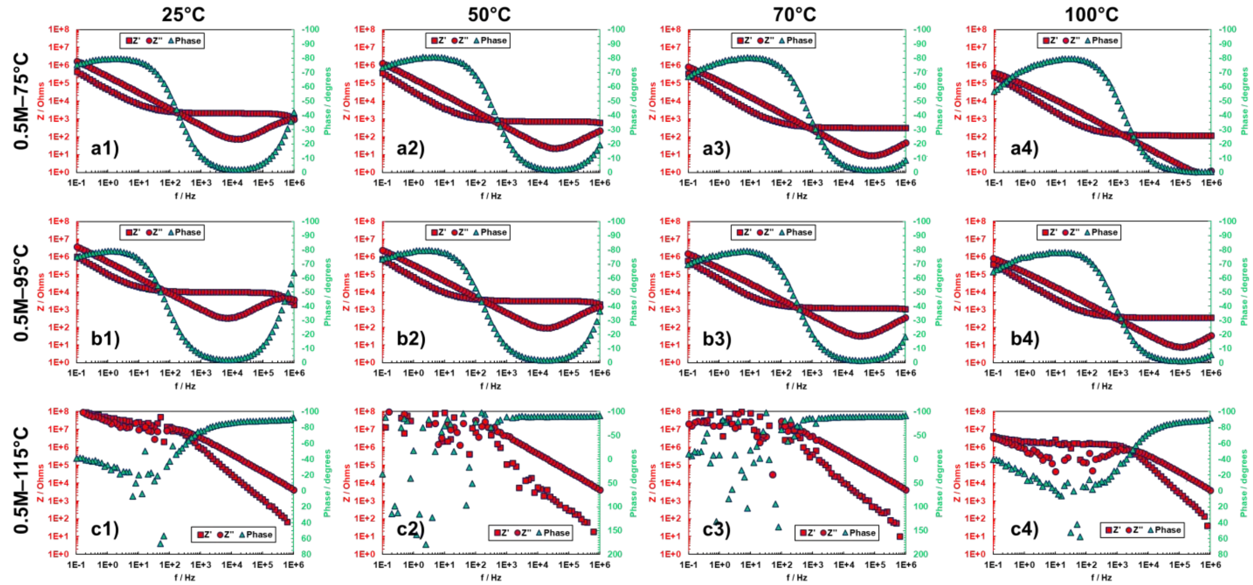

Figure S4. Representative Bode plots for different drying temperatures (0.5M salt concentration) Rows represent the salt concentration, and columns represent selected temperatures at which the spectra were collected.

Table S1. FTIR peak assignment for NMP solvent

Peak	Assignment
1670 cm^{-1}	ν(C=O)

Table S2. FTIR peak assignments for $Ca(TFSI)_2$ salt

Peak	Assignment
409 cm^{-1}	$\omega(SO_2)$
511 cm^{-1}	$\delta_a(CF_3)$
574 cm^{-1}	$\delta_a(CF_3)$
599 cm^{-1}	$\delta_{in\text{–}plane}(SO_2)$
651 cm^{-1}	δ(SNS)
1057 cm^{-1}	ν_a(SNS)
1136 cm^{-1}	$\nu_{s\ \text{out–of–plane}}(SO_2)$
1203 cm^{-1}	$\nu_a(CF_3)$
1248 cm^{-1}	$\nu_s(CF_3)$

Table S3. FTIR peak assignments for PVDF polymer

Peak	Assignment
472 cm^{-1}	$\omega(CH_2)$
486 cm^{-1}	$\omega(CF_2)$
511 cm^{-1}	$\delta(CF_2)$
532 cm^{-1}	$\delta(CF_2)$
614 cm^{-1}	$\delta(CF_2) + \delta(CCC)_{skeletal}$

762 cm^{-1}	$\delta(CF_2) + \delta(CCC)_{skeletal}$
796 cm^{-1}	$r(CH_2)$
828 cm^{-1}	$r(CH_2) + v_a(CF_2)$
876 cm^{-1}	$v_s(CC) + v_s(CF_2)$
976 cm^{-1}	$CH_{out–of–plane}$ deformation
1071 cm^{-1}	$v_a(CC) + \omega(CH_2) + \omega(CF_2)$
1180 cm^{-1}	$\delta(CCC)_{skeletal} + v_s(CC) + v_s(CF_2)$
1247 cm^{-1}	Possibly $CF_{out–of–plane}$ deformation
1281 cm^{-1}	$v_a(CF_2) + r(CF_2) + r(CH_2)$

References

1. Kobayashi, M., Tashiro, K., & Tadokoro, H. (1975). Molecular vibrations of three crystal forms of poly (vinylidene fluoride). *Macromolecules*, *8*(2), 158-171.

2. Bachmann, M. A., & Koenig, J. L. (1981). Vibrational analysis of phase III of poly (vinylidene fluoride). *The Journal of Chemical Physics*, *74*(10), 5896-5910.

3. Lanceros-Mendez, S., Mano, J. F., Costa, A. M., & Schmidt, V. H. (2001). FTIR and DSC studies of mechanically deformed β-PVDF films. *Journal of Macromolecular Science, Part B*, *40*(3-4), 517-527.

4. Ponzio, E. A., Echevarria, R., Morales, G. M., & Barbero, C. (2001). Removal of N-methylpyrrolidone hydrogen-bonded to polyaniline free-standing films by protonation–deprotonation cycles or thermal heating. *Polymer international*, *50*(11), 1180-1185.

5. Bormashenko, Y., Pogreb, R., Stanevsky, O., & Bormashenko, E. (2004). Vibrational spectrum of PVDF and its interpretation. *Polymer testing*, *23*(7), 791-796.

6. Peng, Y., & Wu, P. (2004). A two dimensional infrared correlation spectroscopic study on the structure changes of PVDF during the melting process. *Polymer*, *45*(15), 5295-5299.

7. Rey, I., Johansson, P., Lindgren, J., Lassegues, J. C., Grondin, J., & Servant, L. (1998). Spectroscopic and theoretical study of (CF3SO2) 2N-(TFSI-) and (CF3SO2) 2NH (HTFSI). *The Journal of Physical Chemistry A*, *102*(19), 3249-3258.
